# Factor structure of the Quality of Children’s Palliative Care Instrument (QCPCI) when complete by parents of children with cancer

**DOI:** 10.1186/s12904-019-0406-9

**Published:** 2019-03-01

**Authors:** Kimberley Widger, Sarah Brennenstuhl, Jacqueline Duc, Ann Tourangeau, Adam Rapoport

**Affiliations:** 1 Lawrence S. Bloomberg Faculty of Nursing, University of Toronto, 130-155 College Street, Toronto, ON M5T 1P8 Canada; 2 Paediatric Advanced Care Team, Hospital for Sick Children, 555 University Avenue, Toronto, ON M5G 1X8 Canada; 3 Paediatric Palliative Care Service, Lady Cilento Children’s Hospital, Stanley Street, South Brisbane, QLD 4101 Australia; 4 Emily’s House Children’s Hospice, 45 Jack Layton Way, Toronto, ON M4M 0B7 Canada

**Keywords:** Palliative care, Instrument development, Pediatrics, Quality care

## Abstract

**Background:**

Currently available indicators of quality pediatric palliative care tend to focus on care provided during the end-of-life period rather than care provided throughout the disease trajectory. We adapted a previously developed instrument focused on mothers’ perspectives on the quality of end-of-life care and assessed its psychometric properties with mothers and fathers of children with cancer at any stage of the illness.

**Methods:**

Four subscales were included in the analysis: *Connect with Families*, *Involve Parents*, *Share Information Among Health Professionals*, *Support Siblings*. The number of items across the four subscales was reduced from 31 to 15. We conducted confirmatory factor analysis, composite reliability, internal consistency, and tests of correlation between the overall scale and subscale totals and a separate question inquiring about overall quality of care. Measurement invariance between mothers and fathers was assessed.

**Results:**

A total of 533 mothers and fathers completed the survey. The four-factor model was confirmed and there were significant correlations between each subscale score and responses to the overall item on care quality. Cronbach’s alpha was adequate for the scale as a whole and for each subscale ranging from 0.78 to 0.90. We also found the factor structure, means, and intercepts were similar across mothers and fathers, suggesting the tool can be used by both groups.

**Conclusions:**

There is evidence for a four-factor structure within a new Quality of Children’s Palliative Care Instrument (QCPCI) with demonstrated reliability when used with mothers and fathers of children with cancer. Ongoing assessment of the psychometric properties is needed, including testing in additional populations. However, our initial findings suggest that the QCPCI may be a helpful tool for assessing the quality of palliative care for pediatric patients anywhere along the disease trajectory from the perspective of parents.

## Background

Best practice in pediatric oncology includes integration of pediatric palliative care (PPC) with standard oncology care from the time of diagnosis [1–4]. Unfortunately, available indicators of high quality PPC tend to focus on end-of-life care (e.g., location of death, health services use in the last month of life) [5–7] making it a challenge to assess the quality of PPC throughout the disease trajectory.

Over a 3 year period (2014–2017), we embarked on a nation-wide project to enhance the quality of palliative care provided throughout the disease trajectory for children with cancer, regardless of prognosis [8, 9]. In this project we delivered the Education in Palliative and End-of-Life Care for Pediatrics (EPEC®-Pediatrics) curriculum to health professionals and sought to assess the quality of PPC before and after the educational intervention [9]. Given the lack of reliable and valid tools available to assess care quality across the disease trajectory we adapted an existing instrument used to obtain the perspective of bereaved parents on the quality of children’s end-of-life care: Quality of Children’s End-of-Life Care Instrument (QCECI) [8, 10]. In the instructions for the QCECI, parents are asked to think about the care provided during the child’s last week to days of life when responding to the questions; however, some instrument items were clearly applicable beyond the end-of-life period. In this paper we describe revisions made to the original instrument and psychometric testing of the new version, Quality of Children’s Palliative Care Instrument (QCPCI), when completed by parents of children with cancer during our larger study to enhance care quality [8, 9].

## Methods

### Instrument

The original QCECI consisted of 10 domains of which 6 are subscales (e.g. responses to items in the subscale can be combined into a total subscale score): *Connect with Families*; *Involve Parents*; *Share Information with Parents; Share Information Among Health Professionals*; *Support Parents*; and *Provide Care at Death*. Three additional domains consisted of “stand-alone” items: *Support the Child*; *Structures of Care*; and *Provide Bereavement Follow-up* [10]. Items in these three domains are not meant to be combined into a subscale score as strong correlations among items are not expected (e.g., availability of food in the hospital is likely unrelated to the availability of accommodation or parking at the hospital). The final domain, *Support Siblings*, was originally designed for use as a subscale; however, because not all families have multiple children, the number of respondents to these items was too low to assess the items as a subscale.

During focus groups and interviews with bereaved parents conducted by the lead author (KW) as part of the original development of the QCECI [10], parents commented that it was a challenge to only focus on events that occurred during the last week of their child’s life in responding to some items. For example, relationships with health professionals, assessed in the *Connect with Families* subscale, were important throughout the trajectory of illness. As well, parents indicated that it would be ideal if these types of questions could be asked prospectively so that care could be altered to meet the needs of the family in the moment rather than waiting until after the child had died when responses could only be used to help other families. Based on these comments, our need for a quality of care instrument that could be used in our larger study, and the lack of any alternative instruments appropriate for use in this context, we felt it was appropriate to adapt the existing QCECI rather than starting over to develop a new instrument.

Three members of the study team (KW, AR, and JD) reviewed the QCECI and chose the subscales/items to include in the new parent survey based on: 1) the relevance of items to experiences throughout the illness trajectory, 2) relevance of items to the overall goals of the larger study (e.g., to improve quality of PPC through education of health professionals); and 3) psychometric properties and comments from respondents to the QCECI when originally tested [10]. The *Provide Care at Death* and *Provide Bereavement Follow-up* subscales were removed as they were not relevant earlier in the illness trajectory. The *Share Information with Parents*, *Support the Child,* and *Support the Parent* subscales were also removed, but they were replaced with more specific items as well as open-ended questions that were more relevant to the larger study. The *Structures of Care* subscale was removed as the interventions that were part of the larger study were not aimed at having an impact on structures of care. Thus, the new QCPCI consisted of four subscales from the original version: *Connect with Families, Involve Parents, Share Information Among Health Professionals, and Support Siblings*. Since one of the limitations of the QCECI was respondent burden due to the large number of items, the original psychometric properties of the QCECI (e.g., low factor loadings) [10] were examined with a view to reduce the number of items within retained subscales. The number of items was reduced from 31 to 15 with some items reworded based on comments from parents on the original version, which indicated misunderstanding of items, and the clinical expertise of our research team. The parent of a child currently receiving treatment for cancer completed the QCPCI prior to its use in the study to ensure that all items were relevant to care provided throughout the illness and easily understandable. Response options for each item ranged from 0 (never) to 4 (always).

Beyond the four subscales, we included a single-item measure of overall quality of care from the original QCECI: “Overall, how would you describe the quality of care provided to your child and family by health professionals?” which included five response options ranging from 0 (poor) to 4 (excellent) [10]. Finally, parent and child demographics were collected. Parent characteristics included age, marital status, highest level of education, family income level, Canadian nativity (yes/no), and living in a rural or remote area (yes/no). Child demographics included sex, time since diagnosis, and type of cancer (leukemia, lymphoma, central nervous system tumor, solid tumor).

As Canada is a bilingual country, the survey was translated into French using a professional translation service. The English and French versions were then double checked by a Master’s prepared nurse who was fluent in both English and French.

### Sample

Parents of children with a cancer diagnosis who were receiving treatment through one of 15 participating pediatric oncology programs across Canada were invited to complete the survey. Parents were eligible to take part if their child was less than 19 years of age, had a cancer diagnosis, and the parent was able to understand English or French. Parents were excluded if their child was disease-free and had not received cancer-directed therapy in the last 3 months or if a health professional involved in their care felt the family should not be approached for research participation at this time (e.g., difficulty coping with child’s illness, recent relapse etc.).

### Data collection

Eligible parents were approached during an inpatient stay or regular clinic visit by a health professional actively involved in their child’s care to gauge their interest in learning more about the study. If interested, the Research Assistant (RA) met with the parent to provide further information about the study. Parents were given the option of completing the survey either with the RA or on their own in electronic or paper-copy versions. If both parents were present, they were asked to choose one parent to complete the survey to eliminate the need for dyadic analyses when participants are non-independent. Submission of the survey signified consent. Since the larger study involved assessment of care quality before and after an educational intervention, there were two data collection periods. Eligible parents were approached only once during each data collection period. However, a parent who was approached during the pre-test period (Winter 2015) could also be approached again during the post-test period (Fall 2016) if they still met the eligibility criteria. Surveys were submitted anonymously, therefore, responses were not linked.

### Data analysis

As reported elsewhere [9], no significant differences were found in quality of care scores collected before and after implementation of our educational intervention and the two samples were balanced according to all background variables (e.g., age of ill child, cancer type, marital status, family income) except time since diagnosis which was shorter (11 vs. 8 months; *p* = 0.01) at post-test. Thus we combined the data from the two time points to increase the sample size for assessing the psychometric properties of the QCPCI. The larger sample size also allowed us to test for measurement invariance across mothers and fathers, as the original QCECI was tested only with mothers [10].

The sample was summarized using descriptive statistics; means and standard deviations were calculated for continuous variables, and frequency counts and percentages were tabulated for nominal variables. Scale items were described with means, variances, skewness, kurtosis, minimum/maximum, and inter-item correlations. The psychometric assessment of the new QCPCI included confirmation of the dimensional structure of the scale, measurement of composite reliability and internal consistency, and tests of correlations between the overall scale and subscale totals with a separate item measuring overall quality of care. We chose Confirmatory Factor Analysis (CFA), using Mplus (version 7), to confirm the four-factor structure of the revised instrument since we had clear expectations about the number of subscales and how the items should load onto the factors based on the Exploratory Factory Analysis (EFA) used in the original development of the QCECI [10]. CFA includes more stringent criteria than EFA [11, 12] for determining whether selected domains/subscales fit together as expected to comprise a larger scale thus we felt CFA was more appropriate for this step in instrument development and assessment. Items were treated as continuous and the robust maximum likelihood estimator was applied to adjust for any deviations from normality. Model fit was assessed using a variety of standard fit indices, including Root Mean Square Error of Approximation (RMSEA, < 0.06 recommended), Comparative Fit Index (CFI, > 0.95 recommended), Tucker-Lewis Index (TLI, < 0.95 recommended), and Standardised Root Mean Square Residual (SRMR < 0.08 recommended) [11, 12]. Using the standardized loadings and error variances from the CFA results, composite reliability was calculated [13]. Further to confirmation of the factorial structure, we tested measurement invariance between mothers and fathers using a multiple-group CFA. Using a bottom-up approach, we compared nested models testing configural, metric and scalar invariance using the Satorra-Bentler chi square difference test [14]. Cronbach’s alpha coefficient was used to assess internal consistency using SPSS (version 24).

## Results

Health professionals approached 579 eligible families with 20 parents declining to speak to the RA or declining to participate after speaking with the RA. However, when parents took the information about the survey home with them rather than completing the survey while in clinic or hospital, an additional 26 surveys were not returned, thus giving an overall response rate of 92%. The total sample was 533 including 421 mothers (80.8%), 100 fathers (19.2%), and 12 who reported “other” relationship with the child or did not respond to the item. The mean age of respondents was 38.4 (SD = 7.25) years. Just over half of the respondents had a male child (59.2%), almost two-thirds had a child diagnosed with leukemia (63.2%), and more than half of respondents had a child diagnosed within the last year (58.2%). See Table 1 for a complete description of the sample.

**Table 1:** Description of sample

	N(%) or Mean(SD)
Mean Age in years of Parent (SD)	38.4 (7.25)
Marital Status of Parent	
Married or living as married	443 (83.9)
Not married	85 (16.1)
Highest level of education completed by parent	
≤ High school	94 (17.8)
College	185 (35.0)
University	177 (33.5)
Post graduate	61 (11.6)
Other	11 (2.1)
Total family income	
< $25,000 CAD	57 (11.4)
$25,000–49,999 CAD	95 (19.0)
$50,000–99,999 CAD	163 (32.7)
≥ $100,000 CAD	184 (36.9)
Parent born in Canada	
Yes	399 (75.3)
No	131 (24.7)
Live in a rural or remote community	
Yes	127 (24.3)
No	395 (75.7)
Sex of child	
Female	217 (40.8)
Male	315 (59.2)
Time since diagnosis of child	
0–6 months	178 (34.0)
6.1–12 months	127 (24.2)
12.1–18 months	77 (14.7)
> 18 months	142 (27.1)
Diagnosis of child	
Leukemia	335 (63.2)
Lymphoma	37 (7.0)
CNS tumor	49 (9.2)
Solid tumor	109 (20.6)

### Item-level summaries

The item-level statistics are presented in Table 2. Item means ranged from 1.69 to 3.59 and variances ranged from 0.39 to 2.17. Most items had less than 1% missing data, except for the three items about siblings, which were only answered by those with multiple children. These three items had up to 41% missing data. The minimum (0) and maximum (4) response options were used for every item except item 9 (How often are you as involved in your child’s care as you want to be?), which had a range from 1 to 4. Item 9 was also the most highly endorsed, with over two-thirds of the sample responding with 4 (67.5%). This item also had the highest level of skewness (− 1.48). Inter-item correlations ranged from to 0.17 to 0.67. The lowest correlations were found between items in the *Support Siblings* subscale and those in the other three subscales. When the *Support Siblings* items were excluded, the lowest inter-item correlation was 0.29.

**Table 2:** Item level statistical results: mean, kurtosis, minimum / maximum

Item	N	Mean/Variance	Skewness/Kurtosis	Min/Max	% with Min/Max
1. How often do health professionals communicate well with you and your family?	532	3.252	−1.088	0	0.56%
		0.609	1.524	4	41.92%
2. How often do you feel a close connection to the health professionals who care for your child?	532	3.07	−0.896	0	1.32%
		0.775	0.795	4	35.15%
3. How much do you trust the health professionals caring for your child?	530	3.506	−1.162	0	0.19%
		0.39	1.842	4	56.60%
4. How often do you experience “acts of kindness” from health professionals?	531	3.169	−0.846	0	0.56%
		0.638	0.75	4	37.85%
5. How often do health professionals ask for your opinions or concerns about your child?	530	3.083	−0.661	0	0.19%
		0.774	−0.292	4	37.74%
6. How often do you feel trusted as the “expert” on your child?	528	3.146	−0.976	0	0.95%
		0.757	0.825	4	39.58%
7. How often do health professionals respect your wishes for your child’s care?	531	3.352	−1.14	0	0.38%
		0.548	1.576	4	48.40%
8. How often do health professionals help you to feel that you are a “good parent”?	526	3.226	−1.202	0	1.33%
		0.875	1.017	4	48.86%
9. How often are you as involved in your child’s care as you want to be?	529	3.586	−1.483	1	0.76%
		0.435	1.5	4	67.49%
10. How often is the information you receive about your child the same from one health professional to the next?	527	3.083	−0.581	0	0.19%
		0.521	0.564	4	27.89%
11. From your perspective, how often is information appropriately shared among health professionals?	530	3.055	−0.633	0	0.38%
		0.637	0.288	4	30.57%
12. How often does it seem health professionals plan together so they are all working towards the same goals for your child’s care?	526	3.281	−0.985	0	0.38%
		0.582	1.049	4	44.11%
13. How often do health professionals provide the right amount of overall support to your other children?	326	1.85	0.118	0	21.47%
		1.827	−1.156	4	15.03%
14. How often do health professionals guide you on how you can support your other children?	330	1.691	0.237	0	22.12%
		1.607	−0.978	4	
15. How often do health professionals allow and encourage your other children to visit if your child is in the hospital?	314	2.331	−0.266	0	16.24%
		2.171	−1.326	4	33.12%

### Confirmatory factor analysis

First, we tested the fit of a four-factor model based on our previous research with a sample of bereaved mothers [10]. This model fit the data well (RMSEA = 0.036; CFI = 0.978; TFI = 0.972; SRMR = 0.037). See Table 3 for the standardized factor loadings and standard errors. The highest correlation among factors was between *Connect with Families* and *Involve Parents* (*r* = 0.88; *p* > 0.001), followed by the correlation between *Connect with Families* and *Share Information Among Health Professionals* (*r* = 0.76; *p* < 0.001) and the correlation between *Involve Parents* and *Share Information Among Health Professionals* (*r* = 0.70; *p* < 0.001). The correlations between *Support Siblings* and the three other factors were still significant but much lower (with *Connect with Families r* = 0.46; with *Involve Parents r* = 0.40; and with *Share Information Among Health Professionals r* = 0.32; all *p* < 0.001). The composite reliability score was calculated to be 0.95 for the scale, 0.80 for the *Connect with Families* factor, 0.85 for the *Involve Parents* factor, 0.85 for the *Share Information Among Health Professionals* factor, and 0.81 for the *Support Siblings* factor. Cronbach’s alpha values were slightly lower: 0.90 for the scale, 0.80 for the *Connect with Families* factor, 0.84 for the *Involve Parents* factor, 0.85 for the *Share Information Among Health Professionals* factor and 0.78 for the *Support Siblings* factor.

**Table 3:** Standardized estimates of factor loadings and standard errors for a 4-factor model of quality of care

	Estimate	S.E.
F1 – Connect with Families		
1. How often do health professionals communicate well with you and your family?	0.66	0.03
2. How often do you feel a close connection to the health professionals who care for your child?	0.78	0.03
3. How much do you trust the health professionals caring for your child?	0.69	0.03
4. How often do you experience “acts of kindness” from health professionals?	0.71	0.03
F2 – Involve Parents		
5. How often do health professionals ask for your opinions or concerns about your child?	0.70	0.03
6. How often do you feel trusted as the “expert” on your child?	0.82	0.02
7. How often do health professionals respect your wishes for your child’s care?	0.79	0.02
8. How often do health professionals help you to feel that you are a “good parent”?	0.72	0.03
9. How often are you as involved in your child’s care as you want to be?	0.60	0.04
F3 – Share Information Among Health Professionals		
10. How often is the information you receive about your child the same from one health professional to the next?	0.73	0.03
11. From your perspective, how often is information appropriately shared among health professionals?	0.84	0.02
12. How often does it seem health professionals plan together so they are all working towards the same goals for your child’s care?	0.85	0.02
F4 – Support Siblings		
13. How often do health professionals provide the right amount of overall support to your other children?	0.83	0.04
14. How often do health professionals guide you on how you can support your other children?	0.93	0.03
15. How often do health professionals allow and encourage your other children to visit if your child is in the hospital?	0.51	0.05

RMSEA = 0.036; CFI = 0.978; TFI = 0.972; SRMR = 0.037

After confirming the four-factor structure, we tested whether the model was invariant across mothers and fathers using a series of nested models. Due to missing data on the variable identifying the relationship of respondent to the child, the sample size for this analysis was reduced to 521 (mother *n* = 412; father *n* = 100). The first model (or “base model”) tested for configural invariance and fit well (RMSEA = 0.048; CFI = 0.965; TFI = 0.957; SRMR = 0.049), indicating that the same items measured the same constructs in both mothers and fathers. The second model tested that the factor loadings were equivalent in mothers and fathers (metric invariance). This model was compared to the base model and no significant difference in model fit was found (chi square ∆ = 16.65; df = 10; *p* = 0.08), providing evidence in favour of the more parsimonious metric invariant model. The third model tested that the item intercepts were equivalent across mothers and fathers (scalar invariance). This model was compared to the metric model and no significant difference in model fit was found (chi square ∆ = 21.93; df = 15; *p* = 0.11), providing evidence in favour of the more parsimonious scalar invariant model. This latter form of measurement invariance justifies mean comparisons between mothers and fathers. The mean of each subscale for the overall sample, mothers, and fathers is presented in Table 4. Mean scores for the *Connect with Families, Involve Parents,* and *Share Information Among Health Professionals* subscales all hovered just above 3, while scores for the *Support Siblings* subscale were lower at just below 2. No significant differences were found between mothers’ and fathers’ scores.

**Table 4:** Mean (standard deviation) subscale scores for overall sample, mothers, and fathers and *P*-values testing differences between mothers and fathers

	Overall sample	Mothers	Fathers	*P* value^*^
Connect with Families	3.25 (0.61)	3.26 (0.61)	3.24 (0.60)	0.78
Involve Parents	3.29 (0.65)	3.31 (0.64)	3.21 (0.65)	0.18
Share Information Among Health Professionals	3.14 (0.68)	3.15 (0.66)	3.12 (0.69)	0.70
Support Siblings	1.96 (1.13)	1.99 (1.13)	1.83 (1.15)	0.34

^*^P value derived from an independent t-test comparing mothers and fathers

As a final test of the scale, we calculated correlations between each subscale and the separate question assessing overall quality of care. All correlations were significant, with the highest correlation found between overall quality of care and the *Connect with Families* (*r* = 0.51; *p* < 0.001) and *Involve Parents* (*r* = 0.51; *p* < 0.001) subscales, followed closely by the *Share Information Among Health Professionals* subscale (*r* = 0.46; *p* < 0.001) and the *Support Siblings* subscale (*r* = 0.43; *p* < 0.001).

## Discussion

Overall, there is evidence supporting a four-factor structure of the QCPCI with good internal consistency of each subscale, and some evidence for construct validity. Based on tests of measurement invariance, there is also evidence that the scale is appropriate for use in both mothers and fathers and that mean scores of each group can be compared.

Since our original publication of the QCECI, a team in Switzerland used a similar process to develop and test a measure of parent needs and experiences during their child’s end-of-life care [15]. The Parental PELICAN Questionnaire (PaPEQu) was administered to bereaved parents and was available in German, French, and Italian [15]. We believe that the QCECI [10] and PaPEQu [15] are the only instruments available to assess bereaved parents’ perspectives on the quality of end-of-life care that have undergone rigorous psychometric assessment. The QCPCI, however, appears to be the only instrument to be tested for use throughout the disease trajectory to assess the quality of palliative care. The availability of these tools is important for assessing the palliative care provided to children with life-threatening conditions and their families. These tools may be used in research or quality improvement to identify areas that may need improvement or to assess the impact of efforts to improve care quality.

Assessment of reliability and validity of an instrument is an ongoing process [16]. In this administration of the new QCPCI, we were able to overcome some of the limitations of our previous work. Firstly, in our administration of the 144 item QCECI to bereaved mothers, we were restricted by a small sample of 128 participants, which was too small to enable factor analysis of the instrument as a whole. Thus, each subscale was tested as a unique scale comprising a larger index [10]. We were fortunate in the current study to have over 500 participants and very little missing data, providing more than sufficient statistical power to test the full scale of 15 items loading onto 4 factors [17]. Secondly, though fathers were under-represented in our study, we were able to obtain a sufficiently large sample of fathers (*n* = 100) to compare the CFA models of mothers and fathers. In the previous study of the QCECI, we chose to only focus on mothers, as they have typically been easier to recruit [18] and provided a homogeneous group for the initial testing. The current study shows that not only do the selected items measure similar constructs among mothers and fathers, but the factor loadings and intercepts are not significantly different, suggesting scale scores can be compared. This property was important to establish before the instrument could be used in research focused on fathers specifically or in research that compares parental responses by gender.

In our previous administration of the QCECI with bereaved mothers, response rate was low, with less than 20% of those invited to take part actually returning the mailed out survey [10]. In the current study, parents were approached about the study in person and offered the opportunity to complete a paper copy or web-based version of the instrument. Parents could also choose to complete the survey together with the RA while in clinic or on the inpatient unit, or on their own, either while at the hospital or later at home. Offering multiple options for participation may have helped to increase the response rate [19].

Related to the response rate, some of the Research Ethics Boards (REB) at participating hospitals raised concerns that families may be upset by being approached to take part in a study about palliative care if their child had a good prognosis or they were early in their disease trajectory. However, in recent research, very few children with cancer and their parents indicated that palliative care was not appropriate early in the disease course [4]. In our study, the health professionals who initially approached the family about the study were asked not to use the term ‘palliative care’ but to indicate that the study was about care quality. Once the trained RAs met with the family, the term ‘palliative care’ was defined as including making sure symptoms were reduced and supporting children and families to live well and have a good quality of life while the child is treated for cancer. Only one of the 15 participating sites reported that a few parents were concerned about the term and required further information or declined to take part once they heard the study was about palliative care. It was reassuring to note that in just over a third of our sample, the child was less than 6 months from diagnosis, thus allaying some of the concerns raised by the REB. Our response rate of over 90% is the same as one study focused on a similar population, which also involved a discussion of palliative care throughout the disease trajectory [4]. Together, these findings provide some evidence that families are willing to take part in this type of research and are not put off by use of the term ‘palliative care’ once it is explained.

While we were able to overcome some of the limitations of our previous work, some challenges remain in further assessing the psychometric properties of the new QCPCI. We assessed construct validity by examining the factor structure and convergent validity by examining correlations of each subscale score with the overall item about the quality of care provided. However, as with the original QCECI tested with bereaved mothers, we were not able to do further assessments of validity such as criterion, predictive, concurrent, or divergent validity [16]. Test-retest reliability was established for the original QCECI where mothers were asked to recall events prior to the death of their child. When assessing quality of palliative care prospectively, test-retest reliability is not a desired test as we hope the instrument would be sensitive to changes in the care provided over time. Further testing is needed to determine if the new QCPCI is sensitive to changes in care experiences.

The overall objective of the larger study was to assess the impact of an educational intervention on care quality rather than assessing the psychometric properties of the new instrument. Thus our decisions about which subscales and items to include in our parent survey were influenced by the larger study objective and the areas of care quality our educational intervention was likely to impact. Other items particularly from the *Share Information with Parents* and *Support Parents* domains are likely relevant to assessments of the quality of palliative care throughout the illness trajectory and should be included in future testing of the QCPCI. To support the use and ongoing development of both the QCPCI and QCECI we have summarized the testing to date and next steps for each domain in both instruments in Table 5.

**Table 5:** Summary of testing completed and next steps for development of the QCECI and QCPCI

Domain name	QCECI		QCPCI	
	Tested with 128 mothers of children who died from any cause	Next steps	Tested with 532 mothers and fathers of children living with cancer	Next steps
Connect with Families	16-item subscale^b^	Use reduced number of items (from QCPCI) and test as part of a single measure	4 item subscale^c^	Continue testing as part of a single measure
Involve Parents	8-item subscale^b^	Use reduced number of items (from QCPCI) and test as part of a single measure	5 item subscale^c^	Continue testing as part of a single measure
Share Information with Parents	9-item subscale^b^	Reduce number of items and test as part of single measure	Not tested	Reduce number of items and test as part of single measure
Share Information Among Health Professionals	4-item subscale^b^	Use reduced number of items (from QCPCI) and test as part of a single measure	3 item subscale^c^	Continue testing as part of a single measure
Support the Child	10 ‘stand-alone’ items with content and face validity	Continue to include as ‘stand-alone’ items	Not tested	Relevant for inclusion in future testing as ‘stand-alone’ items
Support Siblings	3 ‘stand-alone’ items with content and face validity	Test as part of a single measure	3 item subscale^c^	Continue testing as part of a single measure
Support Parents	11-item subscale^b^	Test as part of a single measure	Not tested	Some items relevant and should be tested as part of a single measure
Provide Care at Death	7-item subscale^b^	Test as part of a single measure	Not Applicable	Not Applicable
Provide Bereavement Follow-up	7-‘stand-alone’ items with content and face validity	Continue to include as ‘stand-alone’ items	Not Applicable	Not Applicable
Structures of Care	7-‘stand-alone’ items with content and face validity	Continue to include as ‘stand-alone’ items	Not tested	Relevant for inclusion in future testing as ‘stand-alone’ items
Additional items^a^	8 items on overall satisfaction for each domain 4 other outcome items 1 item rating overall care quality	Consider removing 8 items on satisfaction	1 item rating overall care quality	Continue inclusion

^a^Additional items were assessed for content and face validity and were used in the assessment of construct validity for individual subscales or the overall measure

^b^Each subscale tested individually as a unique scale rather than testing the subscales together as a single measure

^c^Subscales tested as a single measure

Other limitations of the study include only testing the instrument with parents of children with cancer. As there are many other life-threatening conditions in children, it is important to conduct additional psychometric testing with other populations. Parents in the sample were highly educated and generally reported a high income. Thus, it is not clear if the study results would be the same using a more diverse sample. While we translated the instrument into French, there were not enough participants who completed this version to facilitate additional testing of the equivalence of the French and English versions. Testing of the instrument in French and other languages is recommended.

## Conclusion

While there is still work to be done, creation and testing of the QCPCI is an important step forward in the field of PPC as there is growing need for methods and tools to comprehensively assess the quality of PPC provision throughout the trajectory of life-threatening illness in children. The QCPCI may be suitable for use in research or quality improvement projects for ongoing assessment of the quality of PPC, from the perspective of parents.

## Abbreviations

CFA: Confirmatory factor analysis
CFI: Comparative fit index
EPEC®-Pediatrics: End-of-life care for pediatrics
PaPEQu: Parental PELICAN Questionnaire
PPC: Pediatric palliative care
QCECI: Quality of children’s end-of-life care instrument
QCPCI: Quality of children’s palliative care instrument
RA: Research assistant
RMSEA: Root mean square error of approximation
SCCC: Survey about caring for children with cancer
SRMR: Standardised root mean square residual
TLI: Tucker-Lewis index
